# Successful Outcome Following Repair of Stanford Type A Ascending Aortic Dissection and Sternal Fracture Following Blunt Trauma: A Report of a Rare Case

**DOI:** 10.7759/cureus.109476

**Published:** 2026-05-23

**Authors:** Debashish Nayak, Samarjit Bisoyi

**Affiliations:** 1 Cardiovascular and Thoracic Surgery, Apollo Hospitals Bhubaneswar, Bhubaneswar, IND; 2 Anesthesia, Apollo Hospitals Bhubaneswar, Bhubaneswar, IND

**Keywords:** aortic dissection, aortic surgery, cardiopulmonary bypass, stanford type a dissection, sternal fracture

## Abstract

We present a rare case of post-traumatic ascending aortic dissection along with a sternal fracture. The patient underwent a successful surgical procedure involving excision of the aorta affected by dissection, replacement of the ascending aorta, and repair of the sternal fracture. Although traumatic injuries to the aorta are considered a common cause of death, reports on successful surgery for ascending aortic (Stanford type A) dissection with sternal fracture are scarce. To the best of our knowledge, this is the first such case reported from India, and only a few similar cases of traumatic ascending aortic dissection with solid viscus injuries of rib fractures have been reported from other countries. This case indicates that successful outcomes could be anticipated if diagnosed early and treated aggressively with replacement or repair of the ascending aorta on cardiopulmonary bypass (CPB).

## Introduction

Blunt thoracic aortic injury (BTAI) is the second leading cause of mortality following vehicular accidents or other crush injuries [1]. Such high-impact incidents create extreme stress on the aortic walls that may either rupture the aorta directly or produce an internal tear. Around 80% of patients die before reaching the hospital, and the majority of the remaining fatalities occur within the first hour of hospital arrival [1]. The aortic isthmus is typically involved in 90% of BTAI cases. However, the injury may also affect the ascending aorta/aortic root, aortic arch, and distal descending aorta [2]. According to the Stanford classification, aortic dissection is broadly classified into two types: type A and type B [3]. Type A involves the ascending aorta, whereas type B involves the descending aorta. However, based on the origin and extent of dissection, DeBakey further subdivides the dissection into three categories: type I, type II, and type III [3]. In type I, the dissection begins in the ascending aorta and extends to the aortic arch and descending aorta; type II dissection arises in and is confined to the ascending aorta; and type III arises in the descending aorta and extends distally above or below the diaphragm [3].

Clinical manifestation of BTAI in a patient includes sudden and severe chest pain and back pain, occurring in almost 76%-96% of cases. Additionally, the patient might also experience shortness of breath, loss of consciousness, vision loss, and weakness. In rare cases, such patients might present with atypical or mild to no symptoms at all [3,4]. It has been reported that the prevalence of type A aortic dissection is twice that of type B and is significantly more fatal [3]. However, the survival rate for patients with type A aortic dissection can be improved with early diagnosis and management. Here, we present a rare case of the treatment and management of ascending aortic (Stanford type A) dissection with an accompanying sternum fracture in a 42-year-old man, who arrived at the hospital three days post-incident with severe chest pain and difficulty breathing. A literature review suggested that only a handful of similar case reports have been reported globally [5,6], with no such case reported from India.

## Case presentation

**Figure 1:**
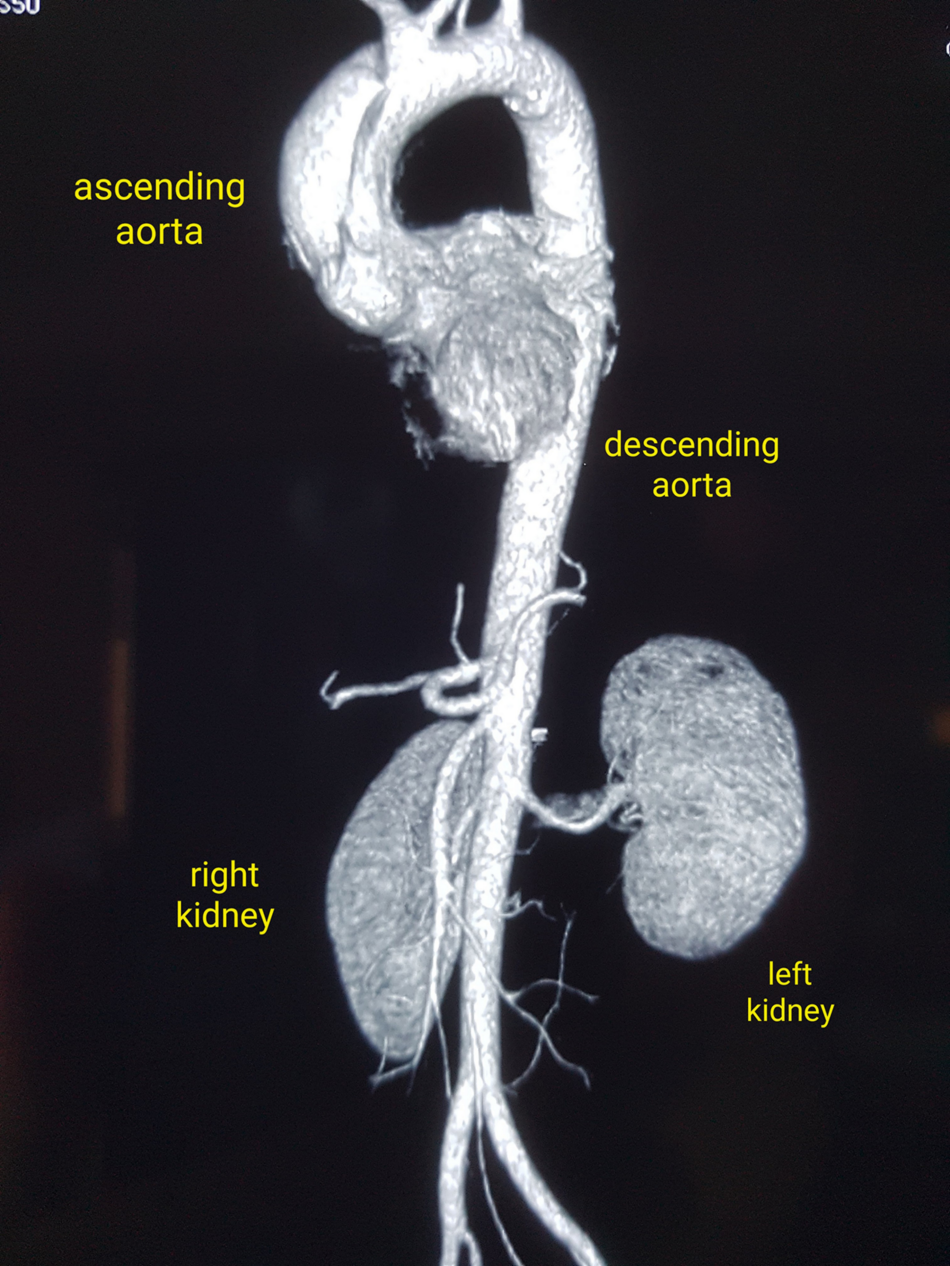
CT aortogram showing dissection in the anterior wall of the ascending aorta

A 42-year-old male patient presented to the emergency department with severe chest pain and difficulty breathing three days after being injured in a road traffic accident (RTA) where his tractor was rolled over. The patient was initially treated at another hospital and then referred to this hospital after the extent of his injuries was realized. He was considered stable enough for transportation, which led to delayed presentation. There was no significant past surgical or medical history. Examination and tests revealed multiple bilateral rib fractures and a transverse sternal body fracture with flail chest and normal blood test results. Routine 2D echo of the heart showed a large dissection in the ascending aorta with a normal aortic valve and a large pericardial collection. An immediate CT angiogram showed dissection of the ascending aorta (Stanford type A/DeBakey type II), distally at the sinotubular junction and extending proximally to the origin of the brachiocephalic artery without involving the arch (Figure 1).

The patient underwent emergency exploration on cardiopulmonary bypass (CPB) as soon as the diagnostic evaluation was completed. The sternum was completely fractured; the manubrium was separated horizontally from the body of the sternum at the angle of Louis. A large hemorrhagic pericardial collection was drained (about 300 mL).

After fibrillating the heart, the aorta was cross-clamped as distally as possible and opened longitudinally. The intima was found dissected anteriorly and laterally with an entry 15 mm above the sinotubular junction in the anterior aspect of the ascending aorta and an exit 10 mm proximal to the ascending-arch junction.

**Figure 2:**
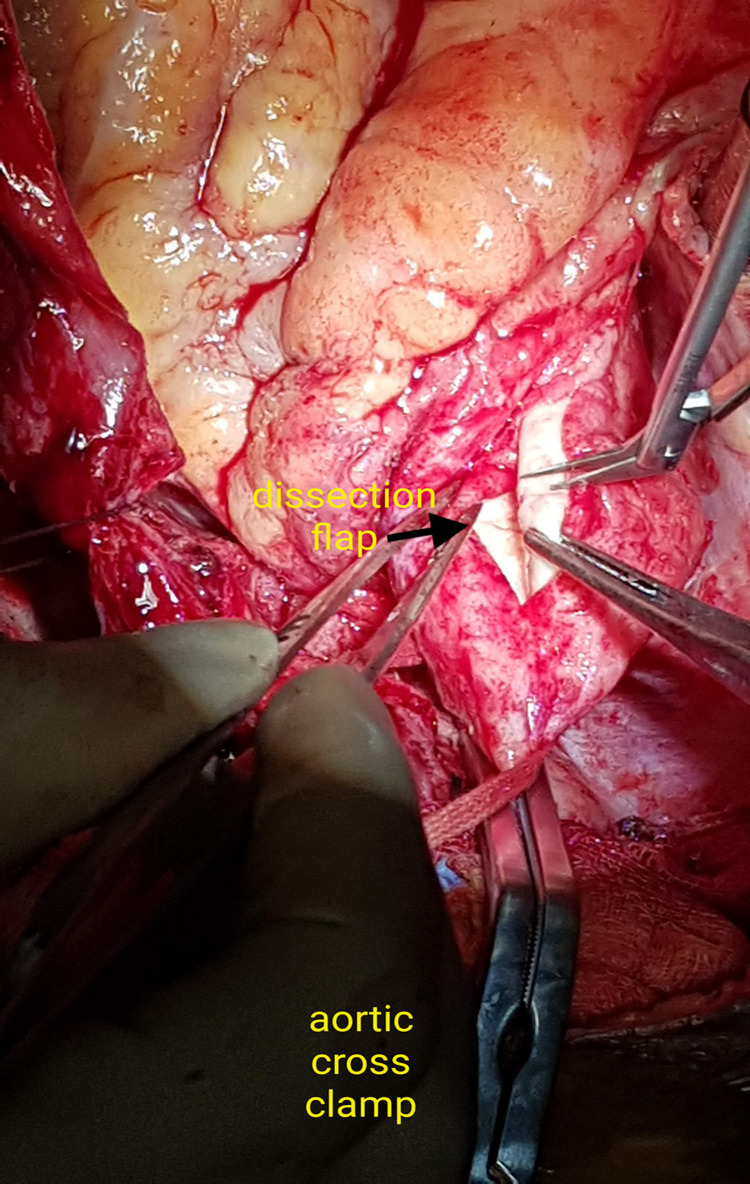
Operative photo showing opening of the ascending aorta with dissecting flap (white, labeled) in situ

**Figure 3:**
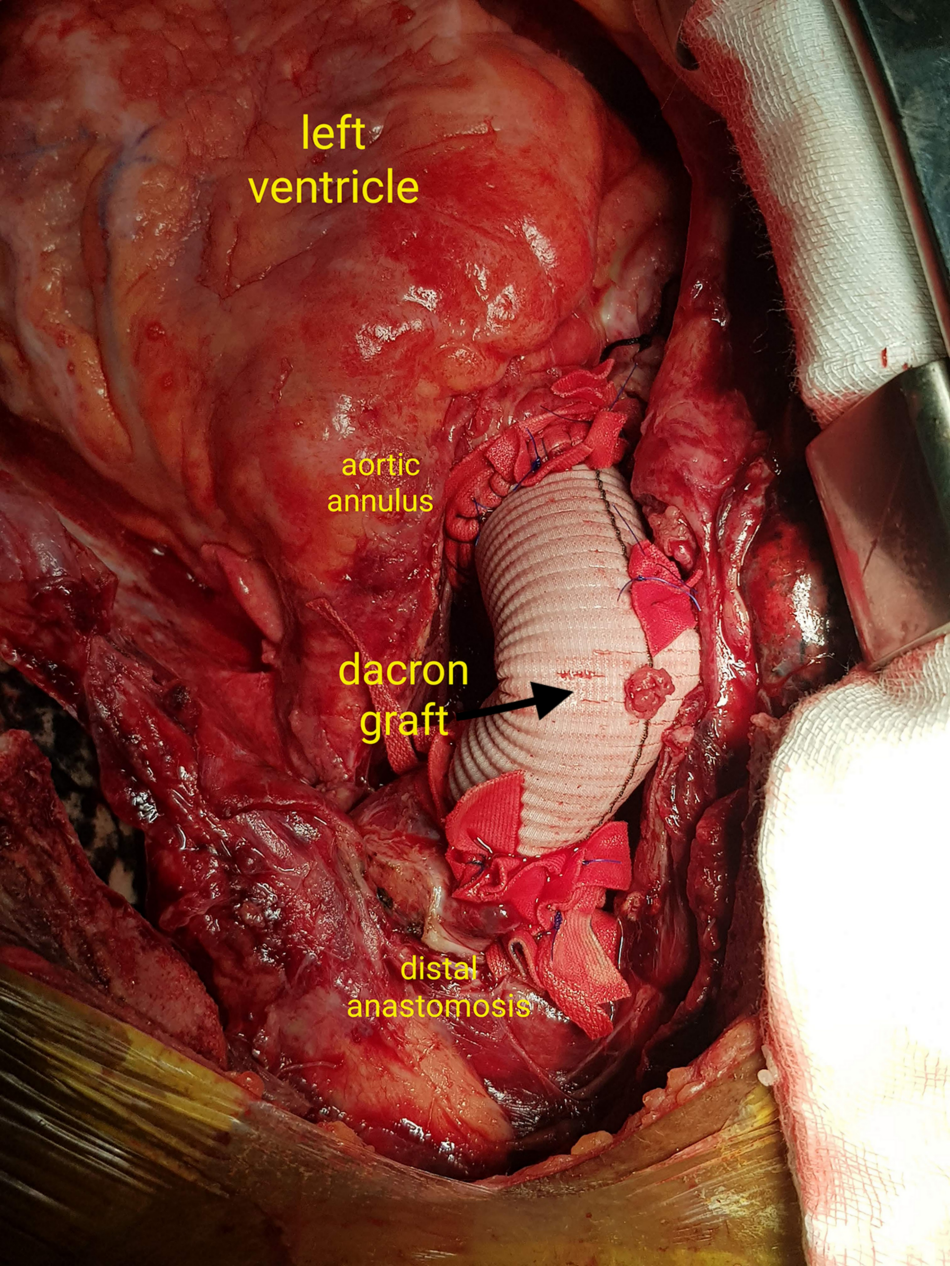
Postoperative photo showing the Dacron graft used in replacing the ascending portion of the aorta (labeled)

The dissection did not extend into the arch of the aorta or coronary buttons, and there was no aortic regurgitation. The intimal flap was opened, and direct ostial cardioplegia was given to arrest the heart (Figure 2). The aorta was dissected all around and excised from just distal to the sinotubular junction to just proximal to the ascending-arch junction. Total circulatory arrest and open repair of the arch of the aorta were not needed since the dissection stopped short of involving the arch. Both ends of the aorta were reinforced by sandwiching the layers of the aortic wall between two layers of polytetrafluoroethylene (PTFE) strip. A commercially available synthetic vascular graft (Dacron tube graft; JOTEC GmbH, Hechingen, Germany) was then cut to size and anastomosed to both ends of the aorta with continuous 4-0 polypropylene sutures reinforced with commercially available glue (Figure 3).

After standard rewarming and de-airing of the heart, the cross-clamp was removed, and cardiopulmonary bypass (CPB) was weaned off easily. The chest was closed in standard fashion with figure-of-eight sternal wires and one commercially available sternal ZIPFIX™ system (DePuy Synthes, Johnson & Johnson, Oberdorf, Switzerland) band after performing the bilateral Robicsek’s repair. The postoperative period of the patient was without complications, leading to extubation on day 1 POD and cessation of inotropes by day 2. He was extubated after two days and shifted to a high dependency unit (HDU) on day 8. After complete recovery, he was discharged on day 14 POD. At discharge, the wound was healing well, and the postoperative 2D echo heart test was normal. The sternum was stable, but mild flail chest was still present.

At three months follow-up, he was normal and asymptomatic (New York Heart Association (NYHA) class I) with a completely healed wound. The sternum was stable. Follow-up blood and 2D echo reports were all normal. He died 13 months post-surgery due to an unrelated cause (suspected cerebrovascular accident).

## Discussion

Dissection of the aorta per section is an emergency condition. Although etiopathogenesis is different for traumatic and non-traumatic aortic dissections, the same guidelines are followed in both treatments [7]. Little information is available about traumatic aortic dissection due to blunt injury and the rarity of survival following such injuries, and consequently, very few reports exist. In general, Stanford type A or DeBakey type II pathology (ascending aortic dissection) has a poor prognosis and causes immediate death in 25%-30% of patients, followed by 1%-2% fatality per hour, leaving only 5% alive at two weeks after the onset of the disease. Usual presentation includes severe chest or back pain, syncope, and shock. Syncope follows malperfusion of the brain; this may result if the arch is involved. In about 30% of cases, distal/peripheral vessels are involved, leading to malperfusion of the affected organ/limb. Tamponade is a common complication. If suspected, the diagnosis is confirmed with an echo of the heart and CT angiogram. 2D echo is the screening test of choice, but a D-dimer assay of more than 500 ngm/dL is also reported in one study as equally accurate in diagnosing aortic dissection [8]. CT angiogram is definitive and a must-do whether surgical or endovascular or medical treatment is being planned or applied [9-13].

Surgery following aortic dissection carries considerable morbidity and mortality. Appelbaum et al. noted in a study conducted between 1966 and 1973 in the USA that the in-house mortality was 88% for patients operated for DeBakey type I or II dissection and 32% for type III [14]. Compared to Wheat’s reported mortality with medical management of aortic dissection, they felt that surgery showed improvement in survival in type I and II dissections and the same results as medical management in type III dissections. These figures have only improved significantly as techniques and technology have progressed. These include deep hypothermic circulatory arrest (DHCA), allowing open distal anastomosis to the arch and complete excision of the dissection flap if in the arch. Vast improvements have also been made in perioperative care; endovascular stenting and the introduction of bio-glue have made an impact. Currently, surgery is the treatment of choice in ascending (type A) dissections, while medical management with careful follow-up or endovascular stenting is the choice for descending (type B) dissections [9-12].

The mechanism of traumatic injury leading to dissection of the aorta, excluding those that are caused by iatrogenic procedures, is well described. Traumatic injuries to the aorta comprise a spectrum called blunt trauma to the aorta (BTA), which is a huge clinical entity and beyond the scope of this article to be discussed. BTA usually occurs (96% of cases) at the junction of the arch-descending thoracic aorta, just distal to the left subclavian artery, called the “aortic isthmus.” This point, where the aorta is attached to the ligamentum arteriosum, is where the fixed part of the aorta (the arch) meets the more mobile part of the aorta (the descending thoracic aorta) and is the usual “torsion point” of the aorta. It gets injured in massive deceleration injuries involving the chest and back by a “water-hammer effect” produced by such injuries. In one post-mortem study series, traumatic aortic rupture (with similar mechanism and distribution as traumatic aortic dissection) was found in 55%-65% of cases in the “aortic isthmus” and 10%-14% of cases in the aortic arch or ascending aorta. Alternate mechanisms of BTA propose that the aorta may be getting injured due to “stretching of the aorta” and a “sudden rise in intravascular pressure” following an injury. Most cases of blunt aortic injuries result from massive deceleration or lateral impact injuries from motor vehicle accidents. BTA involving the ascending aorta, however, usually results from “direct compressive injury” or the so-called “osseous pinch” from sternal/rib fractures due to frontal impact. It is proposed that the ascending aorta is compressed between the sternum in front and vertebral bodies and mediastinal structures behind. Be that as may, why in some cases BTA leads to dissection, and rupture in others, is unknown; traumatic aortic dissection of the ascending aorta is an extremely rare entity and therefore poorly understood. Perhaps lesser degrees of injury result in intimal tears or hematomas, which rupture and then extend proximally or distally, creating a dissecting intimal flap, while more massive injuries cause complete rupture [10,12].

We made use of the internet and PubMed to search for similar case reports using the keywords “ascending aorta, dissection, trauma, surgery, Stanford type A.” Only one case of DeBakey type III (Stanford type B) post-traumatic dissection of the descending aorta being repaired has been reported from India in 2018 [9]. Other identified reports include DeBakey type II with injuries to the liver and bilateral rib fractures reported from Japan in 2018 [5]. A case with delayed surgery after 11 months was reported from the same country in 2015 [6]. There are reports of repair of ascending aortic dissection from Japan in 2006 [15], 2003 [16], 2002 [17], 1998 [18], and 1995 [19], and Turkey in 2002 [20], with successful surgical outcome. Annually, 7,000-8,000 deaths are attributed to BTA in the USA and Canada, emphasizing the fact that it is rare to see a survivor following this injury [11,12]. Interestingly, most of these reports and monographs on BTA have reported road traffic accidents, where the patient was wearing a seatbelt with deceleration injury as a possible mechanism of ascending aortic trauma and not direct compression on the sternum. There is no report of a successful outcome of surgical repair of DeBakey type II traumatic dissection caused by frontal impact and sternal fracture with disruption from anywhere else. This is a unique case of successful repair of post-traumatic dissection of the ascending aorta. What made it even rarer was what occurred after the RTA, and with sternal fracture, rib fractures, and flail chest, the patient presented after a delay of three days and underwent sternal fixation for transection of the body. We could find only a few such cases reported previously, with rare successful outcomes through standard internet/PubMed searches.

## Conclusions

In conclusion, post-traumatic dissection of the ascending aorta is critical and requires prompt management. If suspected, such cases should undergo a CT angiogram immediately, followed by urgent exploration and repair; the ascending aorta should be incised longitudinally, the true lumen should be identified, and after arresting the heart with cardioplegia, the ascending aorta should be excised and replaced with a commercially available prosthetic graft as described. Total circulatory arrest with open distal anastomosis might be required in case the dissection extends into the arch of the aorta. The sternum should be carefully fixed with sternal bands; the standard wiring techniques alone are insufficient. As more such cases are further reported, we shall gain better insights regarding the etiopathogenesis to develop improved treatment modalities.
